# Web-Based Imagery Behavioral Activation (WIMBA): Study Protocol for a Randomized Controlled Trial Testing the Effects, Acceptability, and Feasibility of a Mental Imagery Activity Scheduling Training Delivered Online

**DOI:** 10.32872/cpe.12133

**Published:** 2024-06-28

**Authors:** Max Heise, Sanne J. E. Bruijniks, Fritz Renner

**Affiliations:** 1 Clinical Psychology and Psychotherapy Unit, Institute of Psychology, University of Freiburg, Freiburg, Germany; 2 Department of Clinical Psychology, Utrecht University, Utrecht, The Netherlands; Philipps-University of Marburg, Marburg, Germany

**Keywords:** mental imagery, mental simulation, behavioral activation, activity scheduling, depression

## Abstract

**Background:**

Behavioral activation (BA) is an effective and efficacious treatment for depression. Activity scheduling is the central treatment component of BA and involves planning of potentially enjoyable and rewarding activities. Evidence from non-clinical studies suggests that mental imagery simulations of planned activities can increase motivation and anticipated pleasure for these activities.

**Method:**

We describe a randomized controlled trial testing a mental imagery activity scheduling training delivered online in four weekly sessions (total training duration approximately 90 minutes) in a sample meeting diagnostic criteria of a major depressive episode, as indicated by the Diagnostic Short-Interview for Mental Disorders (Mini-DIPS), and not currently receiving treatment. Participants (N = 140) will be randomized to either mental imagery activity scheduling or a wait-list control condition. Depressive symptoms (BDI-II) and behavioral activation (BADS) are the primary outcomes; BDI-II will be measured at Session 1, Session 4, and at two-week follow-up, BADS at Sessions 1-4 and at two-week follow-up.

**Discussion:**

It is discussed how the expected results may reflect mechanisms and effects of a mental imagery activity scheduling training delivered online in a sample of individuals with depression. Concluding we outline next steps for future research and highlight the potential of this novel treatment for dissemination in the wider community and integration into routine care.

## Highlights

- Preliminary data suggest that mental imagery can be used to enhance behavioral activation (BA).
- We describe a RCT testing the effects of web-based imagery-enhanced BA in a depressed sample.
- Online imagery-enhanced BA is easy to disseminate and may improve depression treatment.

A deficit in reward processing is one of the core clinical features of major depressive disorder (MDD). According to early behavioral models, MDD is associated with a decreased engagement in potentially rewarding activities, which leads to reduced reward experiences and a worsening of mood (Lewinsohn, 1974). A number of studies support this theoretical account. Behavioral and neuroimaging findings have suggested that patients with MDD are hyposensitive to reward and hypersensitive to punishment (Alloy et al., 2016; Eshel & Roiser, 2010). In addition, compared to healthy controls, patients with MDD show a reduced expectation of how rewarding or pleasant a future stimulus will be (Gorka et al., 2014). This phenomenon, also referred to as low anticipation of reward, has been related to less active behavior and less positive affect (Bakker et al., 2017).

One effective psychotherapy that aims to target these deficits in reward processing is behavioral activation (BA; Dimidjian et al., 2006, 2011; for a meta-analysis, see Cuijpers et al., 2023). BA can consist of different procedures, of which activity scheduling (i.e., scheduling activities with the aim to increase positive reinforcement) and skill training (i.e., such as social or problem-solving skills) have received most support (Kanter et al., 2010). Multiple studies on BA procedures have linked these interventions to increases in self-reported levels of behavioral activation and to decreases of depressive symptoms (Stein et al., 2021), and even suggested that behavioral activation may play a role across different types of psychotherapies (Bruijniks et al., 2022). Although research that links BA procedures or self-reported behavioral activation to changes in reward functioning is still scarce (Forbes, 2020; Janssen et al., 2021), some studies did already point to a positive association between reward anticipation and increased activation (Bakker et al., 2017; Dichter et al., 2009) and showed that procedures focused on behavioral activation seem to increase neural activity related to motivation (Mori et al., 2018). However, patients with MDD often suffer from low energy levels (Schuch et al., 2017) and poor motivation (Treadway & Zald, 2011) that can act as a barrier towards engaging in behaviors (i.e., activation) that might facilitate reward experiences. As there is still an urgent need to improve treatments for MDD (Cuijpers et al., 2021), identifying ways to optimize BA procedures to improve reward processing in patients with MDD might be a promising way forward.

Recent pre-clinical studies suggest that BA treatment might be facilitated by combining activity scheduling with mental imagery based procedures (Heise et al., 2022; Holmes et al., 2016; Renner et al., 2019). Mental imagery refers to the multi-sensory experience of information from memory (Kosslyn et al., 2001). Imagery-based procedures have a long tradition in many forms of psychotherapy. Prospective mental imagery involves the simulation of future situations or activities. In non-clinical samples, mental imagery of planned activities has been linked to increased motivation for engaging in these activities (Renner et al., 2019). Mental imagery of positive future events has also been shown to increase the estimated likelihood of future events (Boland et al., 2018) and to decrease depressive symptoms and perceived stress (Marciniak et al., 2024). Recently, we showed that affective mental imagery leads to higher motivation for completing activities compared to neutral mental imagery or no mental imagery, suggesting that an affective mental imagery component may be crucial to enhance motivation. These findings however did not translate to the actual performance of activities, possibly due to a ceiling effect within this non-clinical sample (Heise et al., 2022). In a sub-clinical sample, participants who were instructed to generate positive images showed better performance on a behavioral task compared to participants who were instructed to generate negative images (Pictet et al., 2011). Positive imagery has been shown to modulate early attention allocation towards stimuli associated with the imagined activities (Bär et al., 2023). In sum, these studies suggest that individuals with depression, who typically have lower activation levels compared to healthy controls (Manos et al., 2011; Pinto-Meza et al., 2006), might benefit from prospective mental imagery interventions. Indeed, a recent pilot randomized clinical trial that compared imagery-enhanced BA with a wait-list control condition in a sample of patients with late-life depression confirmed that adding mental imagery to BA is feasible and depressive symptoms decreased more in the imagery BA condition, including at 6-month follow-up (Pellas et al., 2022, 2023). However, in this trial the sample size was small (*N* = 41) and restricted to elderly patients with MDD.

While the effects observed by Pellas et al. (2022, 2023) are promising, there is a clear need for additional studies on the clinical utility of imagery-enhanced BA. Accordingly, we propose to test the effects of an unguided, online-delivered imagery BA intervention in a sample of individuals meeting diagnostic criteria for MDD. The change in study format from telephone-based, as in Pellas et al. (2022), to online-based facilitates easier dissemination, potentially expanding access to an empirically-supported treatment. Furthermore, we believe it is also highly relevant to investigate the role of individual differences for the effectiveness of imagery-enhanced BA. Research suggests that imagery vividness is reduced in persons experiencing symptoms of depression (Holmes et al., 2016). Since the intervention builds on imagery of planned, potentially rewarding activities we aim to investigate if individual differences in the ability to generate reward imagery moderate treatment effects. Likewise, some empirical data suggest that symptoms of anhedonia (Webb et al., 2023) and avoidance tendencies (Nasrin et al., 2017) might limit the effectiveness of BA. By investigating how imagery ability, anhedonia, and avoidance tendencies influence the effectiveness of the imagery-enhanced BA interventions we hope to gain a better understanding *for whom* this intervention works best, paving the way for treatment individualization in the future. Additionally, it may also shed some light on the mechanisms underpinning imagery BA effects, thus enabling potential increases in efficiency by focusing on the ‘active ingredients’.

The present study will test the acceptability, feasibility, and effects of a 4-session imagery BA intervention delivered over the internet on behavioral activation and depressive symptoms in individuals meeting diagnostic criteria for MDD. While results from several studies have already supported the use of BA in online settings (e.g., Mueller-Weinitschke et al., 2023; Potsch & Rief, 2024; Puspitasari et al., 2017; Weitzel et al., 2022), the presented study will be the first to test the acceptability, feasibility, and effects of an imagery-enhanced BA intervention in an online setting. Besides, the present study will include multiple measurements of potential mechanisms of change, thereby providing insights into the relationship between the procedure (imagery-enhanced activity scheduling), potential mechanisms of change (reward anticipation, motivation, and behavioral activation), and outcome (depressive symptoms). In addition, the present study will explore the moderating effects of imagery ability, anhedonia, and avoidance tendencies.

Specifically, we expect to find (1) an increase in behavioral activation, measured with the Behavioral Activation for Depression Scale (BADS; Kanter et al., 2007) weekly from baseline to Session 4 and at two-week follow-up, and (2) a decrease in depressive symptoms, measured with the Beck Depression Inventory-II (BDI-II; Beck et al., 1996) at baseline, Session 4, and at two-week follow-up, for participants in the Imagery BA condition, compared to participants in the wait-list control condition. We expect that this difference between conditions in behavioral activation and depressive symptoms will be present at the end of the intervention (Session 4) and will be maintained at two-week follow-up.

## Method

### Design

The study is a two-arm randomized controlled trial with one active intervention condition (Imagery BA) and one wait-list control condition. Participants in both conditions are invited to complete questionnaires at baseline (Session 1), Weeks 1-3 after baseline (Sessions 2-4) and at follow-up (Week 5 after baseline). The study has been pre-registered (see Heise et al., 2022S).

### Participants

Inclusion criteria are (a) meeting the diagnostic criteria for a current major depressive disorder (MDD) as indicated by the Diagnostic Short-Interview for Mental Disorders (Mini-DIPS; Margraf et al., 2017), (b) a BDI-II score ≥ 14, indicating at least mild levels of depression, and (c) age between 18 and 65 years. Participants will be excluded if (1) they are currently in treatment for a mental health condition (psychotherapy and/or medication), (2) they present high levels of suicidality (as indicated by answers > 1 for item 9 of the BDI-II or the respective section in the Mini-DIPS), or (3) if they are meeting diagnostic criteria for one or more of the following disorders: bipolar disorder, psychotic disorders, or substance dependence.

*Sample Size.* Our aim is to obtain a dataset containing *n* = 140 complete observations, that is, with measurements for all four sessions and follow-up. This sample size was pre-registered (Heise et al., 2022S) and determined a priori using GPower (Faul et al., 2009). We assumed a small effect size (as observed in initial pilot testing), 95% Power, and α = 0.05. To account for attrition as observed in initial pilot testing, in which approximately 26% of baseline participants did not complete the follow-up assessment, we plan to recruit a total of *N* = 192 participants from the general population.

### Procedure

#### Screening Process

To inform potential participants about the present study, advertisements and leaflets will be distributed through various online (social media groups, forums focusing on depression) and offline (general practitioners, psychotherapists, psychiatrists, outpatient clinics, university student counseling services) channels. Anyone interested in the study will be referred to an online screening website, where detailed information about the study’s procedures are provided and informed consent is obtained from interested potential participants. Next, participants complete the BDI-II and screening questions for the Mini-DIPS. If complying with inclusion and exclusion criteria, participants may book an appointment for a telephone interview by choosing a suitable date/time from a list displayed online.

#### Telephone Interview

In the telephone interview, trained interviewers complete the Mini-DIPS including sections on Major Depression and current suicidality as well as any other section identified through the respective screening question. This procedure allows assessing whether diagnostic criteria for inclusion/exclusion diagnoses are met as well as for any other diagnoses covered in the Mini-DIPS.

#### Measurements

Participants complete questionnaires at baseline, Weeks 1-3, and follow-up (Week 5; see Table 1 for a complete list of measures, forms, and sampling points) as part of the respective online sessions. Session invitations will be sent out via email. If participants fail to respond to a given session invitation by accessing the provided link within 24 hours, a reminder message is sent out. The link in the reminder message is valid for another five days; if this period elapses without response, the respective participant is excluded from further participation in the study.

**Table 1:** Measures and Sampling Points

Measure	Screening	Session 1/ Baseline	Session 2	Session 3	Session 4	Follow-up
Primary outcomes						
BADS		x	x	x	x	x
BDI-II	x	x			x	x
Secondary outcomes						
Items on acceptability and feasibility						x
WEMWBS		x			x	x
Activity data						
Activity monitoring form		x		x	x	
Activity ratings			x	x		
Potential mediators (measured as activity ratings)						
Reward anticipation			x	x		
Motivation			x	x		
Potential moderators						
CBAS		x	x	x	x	
FRIS		x			x	
SUIS		x				
TEPS		x				

*Note.* BADS = Behavioral Activation for Depression Scale (Kanter et al., 2007); BDI-II = Beck Depression Inventory-II (Beck et al., 1996); CBAS = Cognitive Behavioral Avoidance Scale (Ottenbreit & Dobson, 2004); FRIS = Freiburg Reward Imagery Scale (https://osf.io/9y64q); SUIS = Spontaneous Use of Imagery Scale (Kosslyn et al., 1998); TEPS = Temporal Experience of Pleasure Scale (Gard et al., 2006); WEMWBS = Warwick-Edinburgh Mental Wellbeing Scales (Tennant et al., 2007).

#### Randomization and Recruitment Stop

Block randomization will be used, where each participant will be randomly assigned to one of two equally sized, predetermined blocks. Randomization is performed using the respective function provided by the survey platform formr (Arslan et al., 2020) used in this online study. Recruitment will stop if either of the following two criteria is met: (a) The number of recruited participants, i.e. participants who have completed at least the baseline questionnaires and have been randomized, has reached *N* = 192 or (b) the number of complete observations, i.e. datasets containing measurements for all four sessions and follow-up, has reached *N* = 140. As a compensation for taking part in the study, participants will receive a 25 € online retailer voucher.

#### Intervention: Imagery BA

##### Session Content

The present study’s intervention consists of four sessions. In Session 1 (duration approximately 20 minutes), participants are familiarized with information about prevalence and symptoms of depression, the general rationale of CBT and, more specifically, of BA. At the end of Session 1, and following standard BA procedures (e.g., Addis & Martell, 2004), participants are instructed to monitor the kind of activities they engage in and how these activities influence their mood during the following week. To this end, participants receive email invitations to complete an activity monitoring form (for details, see below) at 6 pm on Day 3 and Day 5 after completion of the first session. During Session 2 (duration approximately 25 minutes), participants will receive short, standardized, constructive written and personalized graphical feedback on their activity monitoring forms. Next, to facilitate the identification of idiosyncratic and meaningful activities, participants will be asked to identify three meaningful life areas from a list (see Appendix), rank these based on personal significance, and nominate personal values pertaining to each of these areas. Participants will be instructed to choose two activities they would like to engage in over the following week from their highest-ranked life area. In preparation for the following mental imagery tasks, participants complete a standard imagery training exercise (see paragraph ‘imagery training’ below). Participants then proceed to schedule a time and date within the following seven days for each previously chosen activity. Importantly, and differing from standard BA procedures, participants complete a guided mental imagery task presented via audio recording in which participants are instructed to simulate engagement in the respective activity (for further details, see paragraph ‘imagery-enhanced activity scheduling’ below). In Session 3 (duration approximately 35 minutes), participants fill out activity monitoring forms corresponding to their activities scheduled in Session 2 and receive standardized, constructive written and personalized graphical feedback on this. Next, participants choose three activities (+1 compared to Session 2) and once again complete imagery-enhanced activity scheduling for each of these activities. While participants are encouraged to try out new activities, previously chosen activities may also be re-scheduled. In Session 4 (duration approximately 20 minutes), following completion of activity monitoring forms and having received feedback on these, the intervention concludes with a summary of the study’s rationale. Participants are encouraged to continue planning positive activities and are reminded of the follow-up assessment two weeks later.

##### Study Format

**Figure 1:**
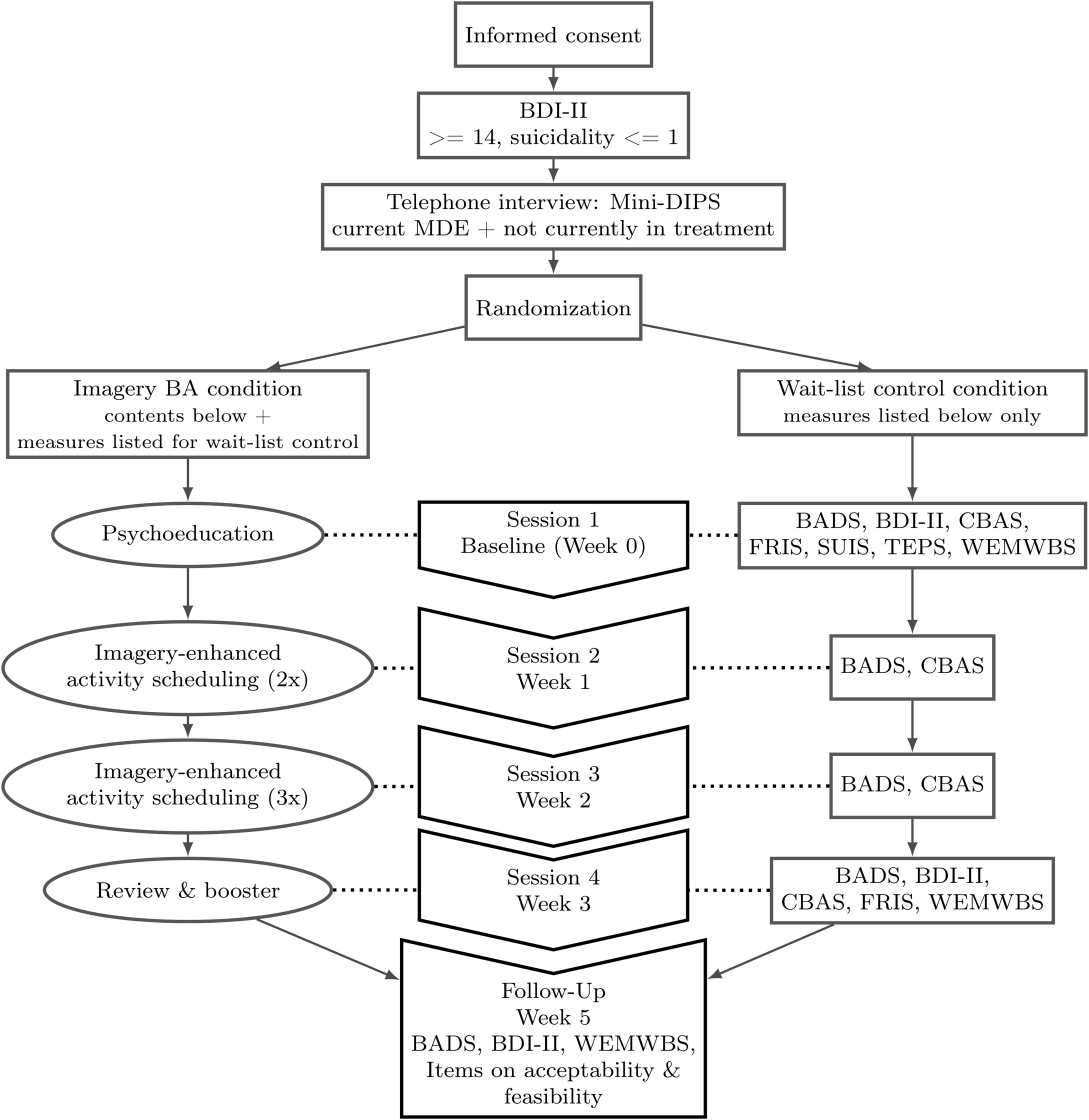
Procedure *Note.* BADS = Behavioral Activation for Depression Scale (Kanter et al., 2007); BDI-II = Beck Depression Inventory (Beck et al., 1996); CBAS = Cognitive Behavioral Avoidance Scale (Röthlin et al., 2010); FRIS = Freiburg Reward Imagery Scale (https://osf.io/9y64q); Mini-DIPS = Diagnostic Short-Interview for Mental Disorders (Margraf et al., 2017); SUIS = Spontaneous Use of Imagery Scale (Kosslyn et al., 1998); TEPS = Temporal Experience of Pleasure Scale (Gard et al., 2006); WEMWBS = Warwick-Edinburgh Mental Wellbeing Scales (Tennant et al., 2007).

Throughout all sessions, content materials are presented on interactive slides using text, pictures, and audio recordings. Apart from the telephone screening interview, the present study is conducted completely online and unguided. However, participants are encouraged to contact the study team if questions or problems related to the study arise. Participant adherence is encouraged through personalized graphical feedback on activity completion, reminder messages, and individualization (focus on idiosyncratic values). Furthermore, the study team continuously monitors participants’ progress and will contact individual participants if necessary. The current version of the intervention incorporates adaptations made in response to participant feedback received in a pilot trial. For a graphical overview of the study’s procedure, see Figure 1.

##### Imagery-Enhanced Activity Scheduling

In the present study, standard BA activity scheduling is enhanced by adding a guided mental imagery task, in which participants are instructed to generate emotionally rich, multisensory mental imagery of how they engage in their previously chosen rewarding activity. Participants are guided through the consecutive stages of initiating, engaging in, and completing the activity. Throughout the latter two stages, emphasis is placed on experiencing the positive emotional impact of the activity. Finally, participants are instructed to create a ‘mental snapshot’ of the positive emotional consequences attributed to the activity. The standardized script used in the present study was based on previous studies (Heise et al., 2022; Renner et al., 2019), adapted to the requirements of the present online study format, and pilot-tested. The audio recordings used for the guided imagery task last 2:51 min (female voice) and 2:46 min (male voice) respectively.

##### Imagery Training

Prior to engaging in the imagery-enhanced activity scheduling for the first time, participants in the Imagery BA condition will complete a standard imagery training task (cf. Holmes & Mathews, 2005). In this training task, participants are instructed to generate vivid, first-person mental imagery while making use of all sensory modalities (vision, sound, smell, and so forth). The audio recordings used for the imagery training last 2:35 min (female voice) and 3:00 min (male voice) respectively.

#### Wait-List Control Condition

For participants in the wait-list control condition, the number and frequency of sessions is identical to those of participants in the Imagery BA condition. However, session contents are restricted to the collection of questionnaire data only. Participants in the wait-list control condition can choose to complete the Imagery BA intervention starting one day after the follow-up assessment.

### Materials

The present study has been implemented on the formr platform (Arslan et al., 2020). This online survey platform will be used to collect questionnaire data, perform block randomization of participants, distribute the audio-visual session contents (including guided imagery scripts) to participants, send out email invitations, and reminder messages. To take part in this study, participants require access to a digital device with internet access and the ability to playback audio files (e.g., smartphone, tablet, or laptop).

### Measures

#### Primary Outcomes

##### Behavioral Activation

Behavioral activation will be assessed weekly from baseline (Session 1) to Session 4, and at two-week follow-up using the Behavioral Activation for Depression Scale (BADS; Kanter et al., 2007; German version: Teismann et al., 2016). The BADS conceptualizes behavioral activation as comprising four distinct factors (activation, avoidance/rumination, work/school impairment, social impairment) measured by asking respondents to indicate agreement with 25 statements (e.g., “I did something that was hard to do but it was worth it.”) on a seven-point scale ranging from 0 “not at all” to 6 “completely”. Reliability and validity of the BADS have been supported (Teismann et al., 2016).

##### Depressive Symptoms

Depressive symptoms will be assessed at baseline (Session 1), Session 4, and at two-week follow-up using the Beck Depression Inventory-II (BDI-II; Beck et al., 1996). The BDI-II measures depressive symptom severity across 21 symptoms by letting respondents choose from four (for two symptoms, seven) statements (e.g., “I blame myself all the time for my faults.”) ranked by increasing severity (scoring 0-3 points per symptom). Total scores can range from 0 to 63, with 0-13 indicating minimal depression, 14-19 mild depression, 20-28 moderate depression, and 29-63 severe depression. Reliability and validity of the BDI-II are well supported (Kühner et al., 2007; Wang & Gorenstein, 2013).

#### Secondary Outcomes

##### Acceptability and Feasibility

Acceptability and feasibility of the newly developed online imagery BA intervention tested in this study will be measured by dropout rate during the intervention and by a number of questions on acceptability and feasibility (e.g., “I liked the online format of the study”, “I found it difficult/unpleasant to engage with the study.”, “I would recommend the training to friends”). Responses will be recorded on seven-point Likert scales with end points labelled 0 “not at all” and 6 “very much” and will be obtained at follow-up or – in the case of drop-out – via an additional questionnaire sent to dropped-out participants. Furthermore, open-ended questions are included to encourage participant feedback on this newly developed intervention.

##### Monitoring of Potential Adverse Effects

Potential adverse effects of the intervention will be assessed in terms of symptom deterioration (BDI-II) as well as through questions on acceptability and feasibility. To minimize the occurrence of adverse effects such as suicidal thoughts and behaviors, extra care is taken to identify persons at risk during the screening procedures and intake telephone interview (for details see inclusion criteria in the Participants section above). In addition, a safety protocol to deal with suicidal ideation that includes consultation with a clinical psychologist has been implemented.

##### Mental Well-Being

Mental well-being will be assessed at baseline, Session 4, and follow-up using the Warwick-Edinburgh Mental Wellbeing Scales (WEMWBS; Tennant et al., 2007; German version: Lang & Bachinger, 2017). Respondents rate frequency of occurrence for 14 statements (e.g., “I’ve been feeling confident.”) during the past two weeks. Answers are recorded on a five-point scale ranging from 1 “none of the time” to 5 “all of the time”. Reliability and validity of the WEMWBS has been confirmed in a German speaking sample (Lang & Bachinger, 2017).

#### Activity Data

##### Activity Ratings

To assess individual differences and changes in reward anticipation and motivation, participants in the Imagery BA condition will be asked to provide ratings of the following items pre and post intervention, that is, before and after the imagery-enhanced activity scheduling procedure: (a) how pleasant they expect the activity to be, (b) how rewarding they expect the activity to be, and (c) how motivated they are to engage in the activity. To control for potential differences in activity characteristics, ratings of activity importance, procrastination tendency, and previous engagement with the activity will be obtained pre intervention. As a manipulation check, allowing to infer whether participants succeeded in generating mental imagery, participants in the Imagery BA condition will rate mental imagery vividness and anticipatory pleasure (i.e., pleasure experienced while imagining the activity) post intervention.

##### Activity Monitoring Forms

To track whether and how participants engage in (scheduled) activities, they are asked to fill out activity monitoring forms in the week between Sessions 1 and 2, as well as in Sessions 3 and 4. In these forms, participants indicate which activities they engaged in (week after Session 1) or whether they engaged in their scheduled activities (Sessions 3 and 4). While inviting participants to fill out the activity monitoring form during the week, as implemented between Sessions 1 and 2, more closely matches standard BA procedures, where activity monitoring is typically given as a homework assignment, it was decided to deviate from this procedure after Session 2 to avoid confounding behavioral intervention effects with the potential reminder effect of activity monitoring invite messages sent in between sessions. For each activity, participants note the duration of their engagement in the activity, how enjoyable/rewarding they experienced the activity to be, and to what extent the activity influenced their mood. In case of non-engagement, participants can note a putative reason for this. The activity monitoring form is presented online and uses an interactive format that adapts to participants’ answers by displaying only relevant elements (e.g., depending on the initial answer whether or not participants engaged in a given activity).

#### Potential Moderators

##### Avoidance Tendencies

Avoidance tendencies will be measured from baseline to Session 4 using the Cognitive Behavioral Avoidance Scale (CBAS; Ottenbreit & Dobson, 2004; German version: Röthlin et al., 2010). In the CBAS, respondents rate appropriateness of 31 statements (e.g., “I quit activities that challenge me too much.”) on a five-point scale ranging from 1 “not at all true for me” to 5 “extremely true for me”. Excellent internal consistency of α = 0.92 has been reported for the German version (Röthlin et al., 2010).

##### The Ability to Generate Reward Imagery

The ability to generate reward imagery will be measured at baseline and Session 4 using the Freiburg Reward Imagery Scale (FRIS), a newly developed scale assessing individual differences in the ability to generate mental imagery of future activities including the associated positive emotional outcomes (for details, see pre-registration at https://osf.io/9y64q). For this scale, respondents are instructed to imagine engaging in a rewarding activity and subsequently rate the resulting mental image (subscales include vividness, anticipatory pleasure, anticipated pleasure, and motivation). Respondents rate their agreement with 12 statements (e.g., “This activity would make me happy.”) on an eleven-point scale ranging from 0 “not at all” to 10 “completely”.

##### Everyday Imagery Use

Everyday imagery use will be measured at baseline using the Spontaneous Use of Imagery Scale (SUIS; Kosslyn et al., 1998; German version: Görgen et al., 2016). In the SUIS, respondents rate the appropriateness of 12 statements (e.g., “When I think about visiting a relative, I almost always have a clear mental picture of him or her.”) on a five-point scale ranging from 1 “never appropriate” to 5 “always completely appropriate”. Acceptable (α = 0.72 - 0.76; Nelis et al., 2014) to good (α = 0.83; McCarthy-Jones et al., 2012) reliability has been reported for the English version. For the German version, Cronbach’s alpha has been reported to be lower (α = 0.66 in both studies; Görgen et al., 2016; Heise et al., 2022).

##### Symptoms of Anhedonia

Symptoms of anhedonia will be measured at baseline using the Temporal Experience of Pleasure Scale (TEPS; Gard et al., 2006). In the TEPS, anhedonia is assessed as two subcomponents, namely anticipatory pleasure (10 items, e.g. “Looking forward to a pleasurable experience is in itself pleasurable.”) and consummatory pleasure (8 items, e.g. “I really enjoy the feeling of a good yawn.”). Respondents rate agreement with items on a six-point scale ranging from 1 “very false for me” to 6 “very true for me”. Acceptable reliability indices for the anticipatory (α = 0.74 – 0.81) and consummatory (α = 0.69 – 0.74) subscales have been reported (Ho et al., 2015).

### Statistical Analyses

We will regard *p*-values less than .05 as criteria for statistically significant results. All reported *p*-values will be two-tailed.

#### Hypothesis 1 – Behavioral Activation

To test for differences between the Imagery BA condition and the wait-list control condition in change in behavioral activation across the five measurement points (Sessions 1–4, follow-up), we plan to conduct a repeated-measures ANOVA on the BADS score with condition (Imagery BA vs. wait-list control) as between-subject factor and time (Sessions 1–4, follow-up) as within-subject factor in an intention-to-treat analysis.

#### Hypothesis 2 – Depressive Symptoms

To test for differences between the Imagery BA condition and the wait-list control condition in change in depressive symptoms across the three measurement points (Session 1, Session 4, follow-up), we plan to conduct a repeated-measures ANOVA on the BDI-II score with condition (Imagery BA vs. wait-list control) as between-subject factor and time (Session 1, Session 4, follow-up) as within-subject factor in an intention-to-treat analysis.

### Exploratory Analyses

To assess feasibility and acceptability, we will report the drop-out rate and descriptive data on acceptability and feasibility. Using structural equation modelling that will allow us to model change over time, we will explore whether reward anticipation and motivation mediate the relation between treatment and behavioral activation and treatment and depressive outcome, respectively. Baseline scores on everyday mental imagery use, symptoms of anhedonia, avoidance tendencies, and the ability to generate reward imagery will be tested as potential moderators of the effect of treatment on outcomes (behavioral activation, depressive symptoms).

## Discussion

We presented a study protocol of a randomized controlled trial testing the effects of an online-delivered Imagery BA intervention on behavioral activation and depressive symptoms in individuals with depression. The imagery BA intervention will be compared to a wait-list control group.

While there are promising pre-clinical studies testing the impact of the imagery-enhanced activity scheduling procedure on motivation and reward anticipation (Heise et al., 2022; Renner et al., 2019) and one pilot randomized controlled trial testing the effects of an imagery BA intervention in a sample of patients with late life depression (Pellas et al., 2022), the present study will be the first to test an imagery BA intervention in an online setting with an adequately powered sample of individuals with depression.

This study includes at least two innovative aspects: First, the intervention that is tested in this study combines two evidenced-based therapeutic procedures (BA activity scheduling and mental imagery) into a new intervention. Although BA activity scheduling is an effective intervention in itself, not all patients with depression benefit from activity scheduling and symptoms of depression, such as a lack of energy and loss of pleasure from activities, might be barriers to successful application of activity scheduling. The mental imagery component might offer an opportunity to overcome these barriers by providing a “pre-experience” of the positive aspects of planned activities in the here-and-now while they are planned in. As several studies have shown that BA can be successfully implemented in an online format (for meta-analyses, see Alber et al., 2023; Huguet et al., 2018; recent studies involving online BA include Mueller-Weinitschke et al., 2023; Potsch & Rief, 2024), it will be interesting to see if the same is true for this novel intervention. If confirmed, this would potentially enable broad dissemination to reach individuals with depression who do not have access to other therapy resources or who are on waiting lists for treatment. Also in a face-to-face context, the imagery BA intervention is relatively straightforward to learn and does not require a high degree of training which would potentially foster further dissemination.

The second innovative aspect of the present study is the concurrent measurement of outcome (depressive symptom severity) and the putative mechanism underlying potential changes in this outcome, that is, behavioral activation. In a study testing an internet-delivered BA intervention in a sample of clinically depressed individuals, Fu et al. (2021) found that improvements in symptom severity were mediated and preceded by increases in activation levels. The present study should provide further insights into whether a similar pattern emerges if BA is augmented with mental imagery.

We expect that the imagery BA intervention is not associated with any risks or adverse effects. Previous studies in non-clinical (Heise et al., 2022; Renner et al., 2019) and clinical samples (Pellas et al., 2022, 2023) suggest that the intervention can be delivered in a safe way and is well accepted and tolerated.

One aspect that needs to be considered when interpreting the results of this study is the fact that we plan to compare the intervention to a wait-list control group. Due to the nature of the control condition, we will not be able to test the added effects of the new mental imagery component on top of the effects of BA activity scheduling alone. However, given that this is the first adequately powered trial in this context, starting with a wait-list control condition seems appropriate. Subsequent research should test the added effect of mental imagery simulation in activity scheduling by comparing an imagery-enhanced activity scheduling intervention to a standard BA activity scheduling intervention. Results of pre-clinical work already suggest that the imagery component does have an added value on motivation and reward anticipation for planned activities (Renner et al., 2019) specifically when the imagery component focusses on pre-experiencing pleasant aspects of planned activities (Heise et al., 2022). The added value of the mental imagery component on depressive symptom severity in patients with depression should be tested in subsequent studies.

In conclusion, although a number of evidence-based treatments for depression exist, including behavioral activation treatment, about half of the patients with depression do not get better in treatment and there is room for treatment innovation and improvement (Cuijpers et al., 2021). It is therefore important to explore new procedures to deliver and to amplify established evidenced-based interventions, such as BA activity scheduling. The imagery BA intervention that is tested in the present study is an example of this approach. The results of this study will provide the first empirical evidence of an imagery-enhanced BA intervention in an online setting for individuals with depression.

## Supplementary Materials

The Supplementary Materials contain the pre-registration protocol for the study (see Heise et al., 2022S).



HeiseM.
BruijniksS. J. E.
RennerF.
 (2022S). Web-based imagery behavioural activation (WIMBA) for depression
[Pre-registration protocol]. PsychOpen. https://osf.io/97wuf
10.32872/cpe.12133PMC1130392039119051

## Data Availability

Upon completion of recruitment and data analysis, the data of the presented study will be made available via the Open Science Framework. Alternatively, data will be made available by the authors on reasonable request.

## Appendix: List of Life Areas

- Relationships (family / friends / romantic)
- Sports & outside activities
- Hobbies / creativity / activities alone
- Self-care / recreation / spirituality
- Education & profession
- Everyday tasks

## X Accounts

@_MxHs, @SBruijniks, @Fritz_Renner
